# Changes in sugar-sweetened beverage consumption in the first two years (2018 – 2020) of San Francisco’s tax: A prospective longitudinal study

**DOI:** 10.1371/journal.pgph.0001219

**Published:** 2023-01-25

**Authors:** Lynn D. Silver, Alisa A. Padon, Libo Li, Bethany J. Simard, Thomas K. Greenfield

**Affiliations:** 1 Prevention Policy Group, Public Health Institute, Oakland, California, United States of America; 2 Alcohol Research Group, Public Health Institute, Emeryville, California, United States of America; ICMR-National Institute of Epidemiology, INDIA

## Abstract

**Background:**

Sugar sweetened beverage (SSB) taxes are a promising strategy to decrease SSB consumption, and their inequitable health impacts, while raising revenue to meet social objectives. In 2016, San Francisco passed a one cent per ounce tax on SSBs. This study compared SSB consumption in San Francisco to that in San José, before and after tax implementation in 2018.

**Methods & findings:**

A longitudinal panel of adults (n = 1,443) was surveyed from zip codes in San Francisco and San José, CA with higher densities of Black and Latino residents, racial/ethnic groups with higher SSB consumption in California. SSB consumption was measured at baseline (11/17-1/18), one- (11/18-1/19), and two-years (11/19-1/20) after the SSB tax was implemented in January 2018. Average daily SSB consumption (in ounces) was ascertained using the BevQ-15 instrument and modeled as both continuous and binary (high consumption: ≥6 oz (178 ml) versus low consumption: <6 oz) daily beverage intake measures. Weighted generalized linear models (GLMs) estimated difference-in-differences of SSB consumption between cities by including variables for year, city, and their interaction, adjusting for demographics and sampling source. In San Francisco, average SSB consumption in the sample declined by 34.1% (-3.68 oz, p = 0.004) from baseline to 2 years post-tax, versus San José which declined 16.5% by 2 years post-tax (-1.29 oz, p = 0.157), a non-significant difference-in-differences (-17.6%, adjusted AMR = 0.79, p = 0.224). The probability of high SSB intake in San Francisco declined significantly more than in San José from baseline to 2-years post-tax (AOR[interaction] = 0.49, p = 0.031). The difference-in-differences of odds of high consumption, examining the interaction between cities, time and poverty, was far greater (AOR[city*year 2*federal poverty level] = 0.12, p = 0.010) among those living below 200% of the federal poverty level 2-years post-tax.

**Conclusions:**

Average SSB intake declined significantly in San Francisco post-tax, but the difference in differences between cities over time did not vary significantly. Likelihood of high SSB intake declined significantly more in San Francisco by year 2 and more so among low-income respondents.

## Introduction

It is well documented that sugar sweetened beverages (SSB) contribute to obesity, diabetes, heart disease, cancer, and other illness [1] and to health inequities in these conditions [2,3]. Consumption of SSBs also exhibits a dose-response relationship with all-cause mortality [4]. The unequal burden of diet-driven metabolic disease has also been a contributor to inequitable impact of the COVID-19 pandemic, highlighting the continuing urgency of identifying effective prevention policies [5,6].

Taxation of SSBs is a public health and fiscal policy that has expanded globally over the past decade, now implemented in over 45 countries [7,8]. The policy seeks to respond to the negative health effects of consumption by creating financial incentives to reduce consumption, promote reformulation, and guide consumers to healthier beverage intake. Simultaneously, these fiscal policies have been used to raise revenue for needed social objectives such as food security, improved nutrition, early childhood education and other purposes. In one fiscal year in 7 U.S. cities with taxes, revenue from SSB taxes totaled $134 million, of which 67% was invested in human and community capital and 28% in healthy food access and other health-related investments [9,10].

In the United States, SSB tax policies have been passed by the Navajo Nation (2013), Philadelphia, PA (2017), Boulder, CO (2017), Seattle, WA (2017), and 4 cities in the California “Bay Area”: Berkeley (2013), Oakland (2016), San Francisco (2016) [11] and Albany (2017), CA. An additional tax was passed by Cook County, IL but was repealed after being in effect for 4 months. These taxes varied in design by the size of the tax (ranging from 1 to 2 cents per ounce), inclusion, or exclusion of non-calorically sweetened beverages, and use of revenue. The San Francisco tax applied solely to sugar sweetened beverages with >25 calories per 12 ounces and is a volume-based tax of one cent per ounce applied to distributors, as were all the Bay Area taxes. After Philadelphia, San Francisco, with 875,000 residents, is the largest city currently taxing SSBs in the US. In 2022, SSB taxes continue under debate from Rhode Island to Kazakhstan, highlighting the relevance of ongoing policy evaluation [12,13].

A growing body of evaluation research has emerged, both in the U.S. and globally. The majority of these studies demonstrate partial or complete pass-through of the taxes to retail beverage prices, reductions in their sale or consumption to varying degrees, and reformulation to reduce sugar content where that affected taxation rates [7,14]. In the U.S., using commercial sales data, Silver et al. found a significant 9.6% decline in volume sales of SSBs in the first year after the Berkeley tax [15]. Similarly, using commercial sales data, Léger found a decline of taxed beverages of 14% in Oakland relative to Sacramento over the first year, but 46% of this decrease was offset by border sales [16]. Powell found a 27% decline in SSB volume sales in the first 4 months of the repealed Cook County tax [17]. Roberto et al. found a 26.6% decline in volume sales in Philadelphia in the first year post-implementation of a 1.5 cent per ounce tax when corrected for cross-border shopping [18]. Powell found a significant decline of 22% in volume of taxed beverages sold in the first year of the Seattle 1.75 cent per ounce tax [19]. Studies of individual consumption and of receipts have been less positive. A non-significant 19% decline in taxed ounces consumed was noted in Berkeley’s first year [15], and using an individual respondents’ receipt-based approach, significant reductions at 12 months were not seen in Philadelphia [20]. Regarding the contemporaneous Oakland and San Francisco taxes, Falbe et al. found complete to near complete pass-through of the tax to prices the first year [21]. Cawley and Frisvold studied receipts of households with children in four US cities with taxes, comparing 6 months before and six-month post-tax. They found significant reductions in Philadelphia relative to synthetic controls but no significant reduction in the combined Oakland, San Francisco, and Seattle sample [22].

Fewer studies have longer-term analyses of tax outcomes. In the U.S. Lee et al. found a decline of 0.55 in frequency of SSB consumption per day in low-income communities three years after the Berkeley tax, significantly greater than the comparison areas [23]. Two years after tax implementation, purchases of taxed beverages declined by 42% in Philadelphia compared with Baltimore, and declines were larger amongst shoppers in low-income neighborhoods [24]. Globally, a study of Mexico’s beverage tax reported a 7.6% decline in taxed beverage purchases and a 2.1% increase in nontaxed beverage purchases over 2 years but lacked a control group [25]. A study of a longitudinal cohort in Mexico after three years found increases in SSB non-consumers and reductions in heavy consumers [26]. In Saudi Arabia, after a much larger 50% tax on carbonated drinks started in 2017 and 5% VAT in 2018, volume sales declined by 40.7% after two years relative to pre-tax trends, and 31% compared to untaxed comparison sites [27]. A smaller 10–20% tiered tax in Catalunya implemented in 2017 was accompanied by a 12.1% decline in regular cola purchases over the first two years [28], and a 39% percent decline in prevalence of at least once weekly SSB consumption in 12 to 40 year olds from low-income neighborhoods after one year, compared to a control city [29].

Using a natural experiment, difference-in-differences approach with a longitudinal panel, this study assessed to what extent the modest one cent per fluid ounce (29.6 ml) of SSB tax implemented in January 2018 in San Francisco was associated with reductions in self-reported SSB consumption at one- and two-years post implementation in San Francisco and a control city, and whether effects differed based on race/ethnicity and/or socioeconomic status.

## Methods

The study used a longitudinal survey of San Francisco and San José residents to compare changes in SSB consumption in the first two years after San Francisco implemented the SSB tax on January 1^st^, 2018. An online consent process was employed, and potential respondents were informed that the study was about family nutrition and eating habits to minimize response bias related to SSB-relevant questions. The study was granted a waiver of documentation of consent and approved by the Institutional Review Boards of the Public Health Institute, protocol # I16-024 and ICF Macro, Inc. ICF collected all three waves of data by web survey: baseline (B) just prior to and in the first 24 days of the tax from 12/2017-1/2018, first follow-up (F1) about one-year (10–15 months) post-tax implementation from 11/2018-3/2019, and second follow-up (F2) about two years (22–25 months) post-tax implementation from 11/2019-1/2020. We calculated Euclidean Mahalanobis distances, a method of measuring differences between points in a vector and a mean [30], between standardized city-level measures for San Francisco and potential control cities. U.S. Census data on population size, race/ethnicity, median age, median income, education, and proportion in poverty were compared using Stata’s nnmatch command, and corrected for large-sample bias due to the multiple continuous covariates [31]. San José was the city with the shortest Mahalanobis distances from San Francisco [32,33]. San José is also geographically proximate to San Francisco; the borders are separated by approximately 64 km [34]. Both cities are in California, a state with low overall SSB consumption, and have had similar exposure to other factors reducing consumption such as educational campaigns.

To understand the impact of the tax on those most likely to be at higher risk of health effects, we used a circumscribed sampling frame consisting of the most racially dense neighborhoods within each city, allowing recruitment of more Black and Latino participants, who, while a small part of the population (San Francisco and San Jose were 5.0% and 2.8% non-Hispanic Black, and 15.2% and 31.6% Hispanic, respectively) [35], are often higher consumers of SSBs in the U.S. To achieve the racial/ethnic targets, 9 zip codes in San Francisco and 13 zip codes in San José were selected based on having at least 10% or 4% Black population or 30% or 35% Latino population, respectively. Proportion cut-offs varied according to each city’s specific racial/ethnic make-up. The 9 San Francisco zip codes covered an estimated 65% of the city’s non-Hispanic Black population and 63% of the Latino population [35]. The 13 San José zip codes covered an estimated 67% of the city’s Black population and 55% of the Latino population [35]. In comparison to the overall San Francisco population, the population of the zip codes from which we drew our sample were less likely to be white, more likely to be Latino or Black, and more likely to have low educational attainment [35]. The population of zip codes from which we drew our San José sample were less likely to be Asian compared to the general population of the city [35].

To obtain sufficient sample size to detect modest changes in consumption within budgetary and policy implementation time-sensitivity constraints, and given California’s rapidly declining random digit dial response rates [36], we used a complex sampling design. For the baseline survey, we recruited respondents from 3 sources: mailed address-based sampling (ABS), a web-based non-probability panel (NP), and a panel that was originally recruited via random digit dial (RDD) for an earlier study to assess a 2016 San Francisco SSB warning label law. Response rates from this earlier study were low (46.7% for landline; 12.5% for cell). This led to a shift to mailed address-based sampling pushing respondents to web surveys for this tax evaluation study, while retaining earlier RDD panel respondents. This was complemented with recruitment from a non-probability web panel, whose members reported higher levels of SSB consumption.

Based on Cohen’s power calculation methods, assuming a 5% significance level and 25% attrition of sample over time, an initial sample with 1100 individuals from each city would give an over 98% power of detecting a small interaction effect between intervention and wave on continuous outcome variables (Cohen’s f = .10 or Cohen’s d = .20) [37]. Respondents were eligible if they were (1) aged 18 or older, and (2) residents of San Francisco or San José from within the sampling frame. Questionnaires were completed in English, Spanish and Chinese. In total, 7,424 completed questionnaires were collected, 3,736 in San Francisco and 3,688 in San José, with 2,614 at baseline (B), 2,410 at follow-up time 1 (F1), and 2,400 at follow-up time 2 (F2) (see Fig A in S1 Text). The response rates for repeat respondents, referred to as RR3, represents the percentage of completions among all eligible records in the sample, which was 42% for each of the follow-up surveys. Given the recruitment methods used, assessment of reasons for non-participation was not possible. Respondents were included in the analysis if they participated in B and F1 only (*n* = 198) or B, F1 and F2 (*n* = 1,257). Respondents were then excluded if they ever reported beverage intake greater than 400 ounces (11.8 liters; *n* = 11) or had missing covariates at 2 time points of data, one of which was baseline (*n* = 1). The resulting final analytic sample was 1,443 repeat respondents (SF: n = 722, SJ: n = 721; see Table 1), and based on Hedeker et al, [38] should yield 95% power to detect a small linear interaction between city and time. Respondents who only participated at baseline or were otherwise excluded were more likely to be slightly older, Black or Latino, of lower education (San Francisco only) or economic status (San José only), from the non-probability sample, and have a higher baseline SSB consumption. Due to recruiting challenges, 463 participants in our final sample (21% of the San Francisco sample, 42% of the San José sample) completed the baseline survey within the first 24 days after tax implementation, potentially capturing a baseline beverage intake for SF participants already impacted by the SSB tax, which could underestimate the effect. However, sensitivity analyses excluding them showed no significant differences (Table C in S1 Text), and it therefore appears unlikely that the tax had an effect on beverage intake in the first 3 weeks. With these and sample size considerations, the main analysis was conducted including these participants.

**Table 1:** Characteristics of sample at baseline, overall and by city (2017–2018).

	Total	San Francisco	San José	p–Value^a^
	(n = 1,443)	(n = 721)	(n = 722)	
Age, years, weighted median (IQR)	40 (29–55)	40 (29–55)	40 (29–55)	0.087
	*n (weighted %)*			
Sex				
Male	654 (51.3)	325 (52.2)	329 (50.6)	0.764
Female	789 (48.7)	397 (47.8)	392 (49.4)	
Race/Ethnicity				
White	803 (33.2)	410 (30.2)	393 (35.2)	<0.001
Asian	317 (30.3)	163 (34.3)	154 (27.6)	
Latino	202 (26.5)	75 (23.0)	127 (28.8)	
Black	72 (5.2)	55 (10.7)	17 (1.5)	
Other	49 (4.9)	19 (1.9)	30 (6.8)	
Education				
High School or Less	108 (37.5)	53 (37.8)	55 (37.4)	0.567
Some College	314 (24.8)	138 (22.3)	176 (26.4)	
4–year College	551 (24.3)	290 (26.5)	261 (22.8)	
Graduate or Professional School	470 (13.4)	241 (13.4)	229 (13.4)	
Federal Poverty Level				
Less than 200%	243 (31.4)	140 (37.7)	103 (27.1)	0.043
200% or greater	1,200 (68.6)	582 (62.3)	618 (72.9)	
Sampling Source				
Random digit dialing	462 (33.7)	231 (35.5)	231 (31.1)	0.459
Address–based	569 (38.9)	307 (39.2)	262 (38.3)	
Non–probability	412 (27.4)	183 (25.3)	229 (30.6)	

Note: IQR = interquartile range. “Other race” includes participants who identified as Native Hawaiian or Pacific Islander, American Indian or Alaska Native, or some other race.

^a^p–value of Chi–square test for differences in covariates across cities for all characteristics except age, for which Mood’s median test for differences in medians across cities was conducted.

To correct for respondent attrition and disproportionate sampling probabilities introduced by the sampling design, baseline sample weights were constructed for the final sample and survival probabilities over F1 and F2 were estimated by logistic models. The baseline sample weights were then multiplied by the corresponding survival weights before post-stratification via raking [39] to adjust the baseline sample weights and provide a closer match between the sample and the population across the post-strata defined by city, sex, race, age, and education groups.

## Measures

### SSB consumption

The main outcome was daily sugar-sweetened beverage consumption, measured in ounces (29.6 ml). Usual beverage intake was assessed using the Beverage Intake Questionnaire (BevQ-15) a validated instrument which has correlated well with dietary intake studies [40–42]. The BevQ-15, modified for web administration, estimates mean daily intake of 15 beverage types, including water, 100% juice, tea/coffee, milks, alcohol, diet beverages and SSBs. Consumption of other beverages was ascertained by asking, “Did you drink any other kinds of drinks–for example smoothies, kombucha or horchata?”; up to 5 beverages could be specified. When appropriate, these were reclassified as one of the 15 beverage types (e.g., Champagne classified as alcohol). For each type, participants indicated how many times in the past month they drank the beverage on a 13-category scale. Those who drank the beverage more than once a week were asked how much they drank each occasion using 5 size response categories: “less than 6 ounces (<178ml)”, “8 ounces (237 ml), “12 ounces (355 ml)”, “16 ounces (474 ml)” and “More than 20 ounces (>592 ml).” Responses from the two questions were combined to calculate consumption per day in ounces, using 4 oz (118 ml) as the proxy value for “less than 6 ounces” and 20 oz as the proxy value for “More than 20 ounces,” as recommended by the BevQ developer. Average SSB consumption was determined by summing the estimated daily intake of regular soda, sweet tea, sweetened energy drinks, sweetened juice drinks/ades, and qualifying “other” beverages. In addition to a continuous measure of beverage consumption, a dichotomous measure of high and low daily SSB intake was constructed. High SSB consumption was defined as drinking at least 6 oz (178 ml) of SSBs per day, based on the 75^th^ percentile (5.71 oz/169 ml) of baseline consumption rounded to the nearest ounce, and lower consumption as less than 6 oz. To account for possible non-linearities in the relationship between tax exposure and SSB intake, high SSB consumption was also examined using 4 oz, 8 oz, and 12 oz thresholds (Table D in S1 Text).

### Demographics

Demographic variables included continuous age, sex (male, female), and education (less than or some college versus four-year college or more). Race/ethnicity was dichotomized (white or Asian versus Black, Latino/a, Native Hawaiian or Pacific Islander, American Indian/Alaska Native, or some other race) according to trends in SSB consumption and demographic composition of the study area. In California. white and Asian populations drink fewer SSB’s on average than other racial/ethnic groups [43], and, together, composed over half of the population in San Francisco (white: 41.2%, Asian: 33.5%) and San Jose (white: 27.0%, Asian: 33.5%) during the study period [35]. Federal poverty level, calculated from reported household income range and number of dependents, was categorized into 2 groups (less than 200% versus greater than or equal to 200%). The 200% federal poverty level cut-off was used, because slightly below one third (31.4%) of the weighted sample had household incomes below 200% of FPL, which allowed for comparisons of low versus medium/high socioeconomic status. Due to the stability of federal poverty level between waves, missing values at baseline were imputed with values at F1 for 4 participants, and missing values at F1were imputed with values at F2 for 1 participant.

### Outside-city exposure

The number of days spent in San Francisco in the past 30 days was ascertained of all respondents to capture exposure to San Francisco’s SSB tax regardless of respondent’s place of residence. San Francisco residents were asked, “On how many days in the last 30 days were you outside the City of San Francisco?” and San José residents were asked, “On how many days did you go to the City of San Francisco in the past 30 days?” Number of days spent in San Francisco was calculated as the number of days San José residents traveled to San Francisco in the past 30 days, and the remainder of 30 less the days on which San Francisco residents traveled outside of San Francisco in the past 30 days. The number of days spent in San Francisco was then dichotomized as 16 days or greater (more than half) versus 15 days or fewer (less than or equal to half) spent in San Francisco. Cross-border shopping was measured by asking, “In the past year, have you changed the store or stores where you usually buy your non-alcoholic beverages like soda, juice, water, etc. (not beer, wine, or hard liquor)? If so, did you change to buy more in a store or stores [in/outside of] your current city?”

### Analysis

Weighted frequencies assessed the distribution of sex, race/ethnicity, baseline education, and baseline federal poverty level, overall, by city, by sampling source, and by baseline survey date (prior to January 1^st^, 2018, versus January 1^st^-24^th^). The significance of differences between cities was assessed using Pearson’s chi-2 test of independence. Weighted medians and interquartile ranges of sample age at baseline were determined overall and by city, and the difference between cities was tested using Mood’s median test. Differences across sampling sources and baseline survey date were assessed using the same methods.

### Difference-in-differences GLM analysis 1

Weighted SSB consumption means and 95% confidence intervals (CI’s) were calculated at each time point by city. To detect effects of the SSB tax, we ran a sample weighted generalized linear model (GLM) with a Gamma distribution and a log link and accounted for intra-person correlation using clustered errors to compare the differences in SSB consumption between San Francisco and San José from baseline to years 1 and 2 post-tax implementation. A Gamma GLM was used in light of the right-skewedness, nonnegative nature, and presence of SSB non-consumers in beverage intake measures. The structure of the GLM is below:

$$\ln\left(E\left[SSB_{it}\right]\right)=\beta_{0}+\beta_{1}t_{1}+\beta_{2}t_{2}+\beta_{3}x_{i}+\beta_{4}\left(t_{1}*x_{i}\right)+\beta_{5}\left(t_{2}*x_{i}\right)+\beta_{z}Z_{it}+\varepsilon_{it}$$

SSB_it_ represents beverage intake for individual i at time t. Two dummy-coded time variables representing 1-year (*t*_1_) and 2-years (*t*_2_) post tax, an indicator for respondent’s city (x_i_), and interactions between the two dummy-coded time variables and city were included in the model. We controlled for age, sex, race/ethnicity, education, federal poverty level, and sampling source (represented as *Z_it_* vector above). Estimated marginal means of SSB consumption for both cities at each time point were calculated based on adjusted model results using Stata’s *margins* command [44].

### Difference-in-differences GLM analysis 2

Weighted proportions and 95% CIs of high SSB consumption were calculated at each time point by city. To compare differences in probability of high SSB consumption (6 oz/178 ml or more daily) pre- and post-tax implementation between cities, we modeled the dichotomized high/low SSB consumption outcome using a binomial GLM with a logit link. As before, the model accounted for intra-person correlation using clustered errors and controlled for the same covariates as in the Gamma GLMs. Average predicted probability estimates were calculated based on adjusted model results using Stata’s *margins* command [44]. This model was also used to assess likelihood of change in cross-border shopping pre- and post-tax implementation.

### Difference in-differences across socioeconomic status and race/ethnicity GLM analysis 3

To examine whether difference-in-differences of SSB consumption between cities varied by race/ethnicity or socioeconomic status, the Gamma and binomial GLMs were rerun three times with additional 3-way interactions between the year, city, and one of the following variables: race/ethnicity, educational attainment, and federal poverty level. We examined whether the difference-in-differences of SSB consumption varied by sampling source using the same method.

### Sensitivity analyses

Sensitivity analyses were run using both two-way interaction models, exchanging city of residence with the dichotomized days spent in San Francisco in the past 30 days as the measure of exposure to SSB taxes (<16 days *versus* ≥16 days in past 30 days) to model the cumulative exposure to the SSB tax from pre- to 2-years post tax. One hundred and thirty-one respondents were excluded from the sensitivity analysis (SF: n = 119, SJ: n = 12), because they changed tax exposure categories between year 1 and year 2 post-tax implementation, i.e., they spent 16 days or more in San Francisco at the F1 and fewer than 16 days at F2, or vice-versa. All analyses were performed using Stata 16.1 [45].

## Results

Characteristics of the sample can be seen in Table 1. San Francisco participants were more likely to live under the federal poverty level, more likely to identify as Black, and less likely to identify as another race when compared to San José participants. Across sampling sources, the only statistically significant difference in characteristics was that the ABS sample had slightly higher median age (42 years) than the RDD (38 years) and NP (39 years) samples (Table A in S1 Text). Participants who took the baseline survey prior to January 1, 2018 (tax implementation date) were significantly younger than those who took the survey between January 1^st^ and 24^th^ (median age in SF at baseline: pre-January 1^st^ = 38 versus post-December 31^st^ = 46; in SJ: pre-January 1^st^ = 37 versus post-December 31^st^ = 44). No other significant demographic differences were found between these two groups (Table B in S1 Text).

### Difference-in-differences GLM analysis 1 for SSB consumption

**Fig 1:**
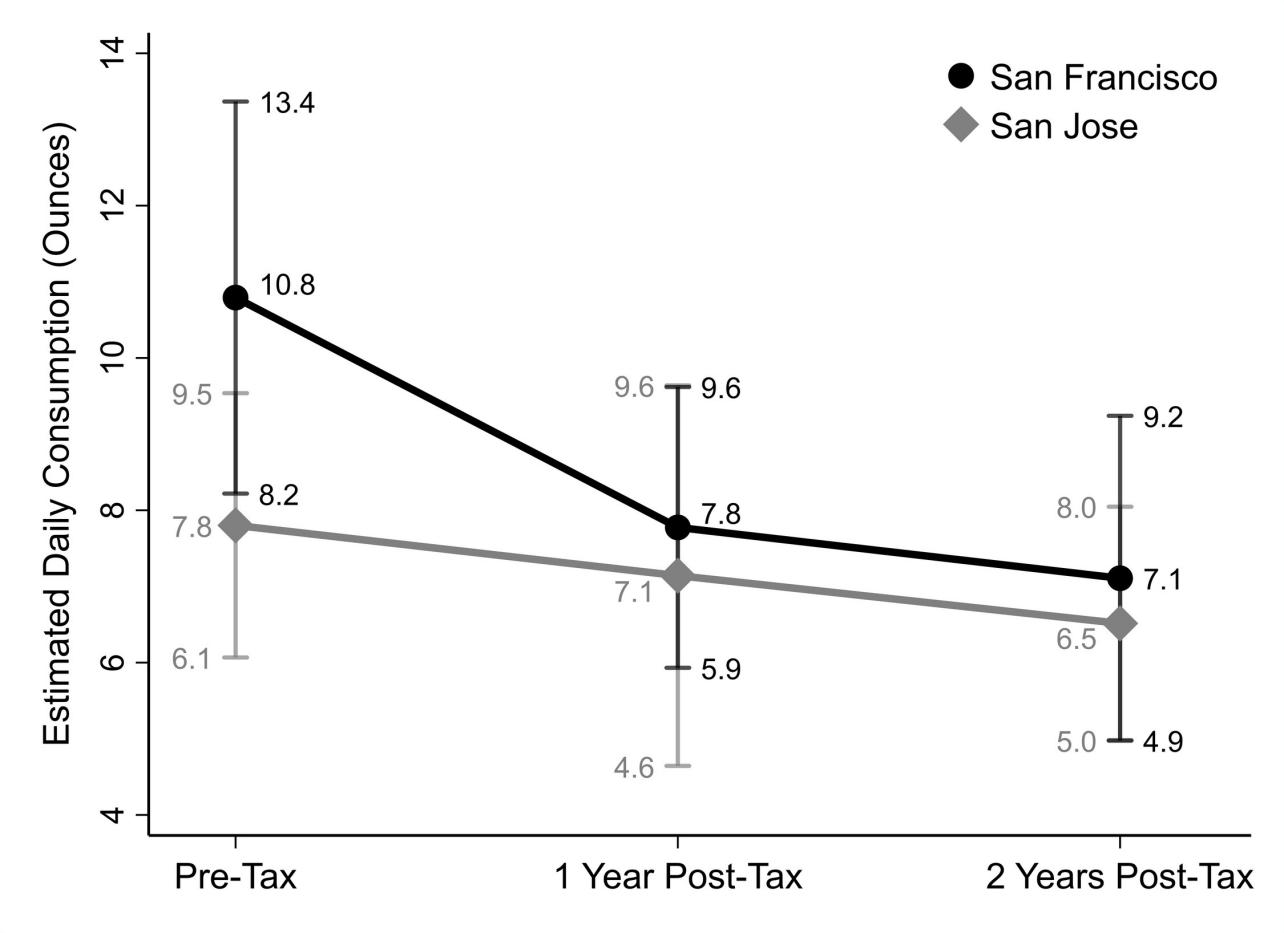
Predicted mean consumption of sugar-sweetened beverages (ounces) with 95% confidence intervals in San Francisco and San José before, one, and two years after San Francisco’s sugar sweetened beverages tax implementation (n = 1,443). Note: Results based on Gamma GLM with 2-way interactions between time (pre-tax, 1-year post-tax, and 2 years post-tax) and city (San Francisco, San José), controlling for age, sex, race/ethnicity, education, federal poverty level and sampling source.

At baseline (B), the unadjusted weighted sample average of daily SSB consumption was 10.87 oz (322 ml) (95% CI: 7.95, 13.78) in San Francisco and 7.26 oz (215 ml) (95% CI: 5.59, 8.93) in San José; one-year post-tax, average consumption had declined to 8.56 oz (253 ml) (95% CI: 6.01, 11.11) in San Francisco and 6.78 oz (201 ml) (95% CI: 4.71, 8.84) in San José; and two-years post-tax, average consumption was 6.42 oz (190 ml) (95% CI: 4.19, 8.65) in San Francisco and 6.32 oz (187 ml) (95% CI: 4.65, 7.98) in San José, a 40.9% decline in San Francisco (-4.45 oz, p = 0.003) and 13% (-0.94, p = 0.397) in San José, a non-significant difference-in-differences of 27.9% between cities between baseline and F2. Table 2 presents unadjusted and adjusted arithmetic mean ratios (AMRs; exponentiated coefficients) and 95% Cis from the Gamma GLM assessing the difference-in-differences in SSB consumption. In the adjusted model, San Francisco residents consumed significantly more SSBs daily, on average, than residents of San José at each time point. While there was a marked downward trend in SSB consumption post-tax that was greater in San Francisco, the difference-in-differences between the cities was non-significant (year 1: adjusted AMR = 0.79, p = 0.308; year 2: adjusted AMR = 0.79, p = 0.224). Predicted mean SSB consumption decreased 2.36 oz more in San Francisco than San José between baseline and 1 year post-tax and 2.40 oz more between pre-tax and 2 years post-taxed. Predicted mean consumption with 95% CIs pre- and post-tax are displayed in Fig 1. In San Francisco, SSB consumption declined by 28.0% from baseline to 1-year post-tax (-3.02 oz, p = 0.001) and by 34.1% at 2 years post-tax (-3.68 oz, p = 0.004). In San José, predicted mean SSB consumption declined by 8.5% 1-year post-tax (-0.66 oz, p = 0.668) and by 16.5% (-1.29 oz, p = 0.157) at 2 years post-tax, a non-significant difference of 17.6% in change over time by year 2 between cities. When excluding the 463 participants who took the baseline survey within the first month of tax implementation, the adjusted Gamma GLM’s yielded results consistent with those of the full sample with respect to the difference-in-difference analysis (Table C in S1 Text).

**Table 2:** Difference-in-differences of sugar-sweetened beverage consumption (ounces) pre- and post-tax implementation between San Francisco and San José pre- and post-tax implementation (n = 1,443).

	Unadjusted Arithmetic Mean Ratio^a^ (95% CI)	Adjusted Arithmetic Mean Ratio^a^ (95% CI)
**Time**		
Pre–Tax	1	1
Year 1 post–tax	0.93 (0.65,1.33)	0.92 (0.61,1.37)
Year 2 post–tax	0.87 (0.63,1.20)	0.84 (0.65,1.07)
**City**		
San Francisco	1.50* (1.05,2.13)	1.38* (1.02,1.87)
San José	1	1
**Difference-in-differences**		
City * Year 1 post–tax interaction	0.84 (0.53,1.35)	0.79 (0.50,1.25)
City * Year 2 post–tax interaction	0.68 (0.42,1.09)	0.79 (0.54,1.16)
Covariates		
Age, years		0.99*** (0.98,0.99)
Sex		
Male		1
Female		0.72* (0.55,0.94)
Race/Ethnicity		
Asian or White		1
Black, Latino, or other race		1.52** (1.14,2.03)
Education		
Some college or lower		1.88*** (1.40,2.52)
Bachelor’s degree or more		1
Federal Poverty Level		
Less than 200%		1.37* (1.05,1.79)
200% or greater		1
Sampling Source		
Random digit dialing		0.49*** (0.35,0.69)
Address–based		0.45*** (0.34,0.60)
Non–probability		1
Constant	7.26*** (5.77, 9.14)	11.93*** (7.30,19.51)

Note

* = p < 0.05

** = p < 0.01

*** = p < 0.001; 1 = reference group; CI = confidence interval.

^a^Exponentiated coefficients of Gamma GLM of SSB consumption in ounces.

### Difference-in-differences GLM analysis 2 for high SSB consumption

**Fig 2:**
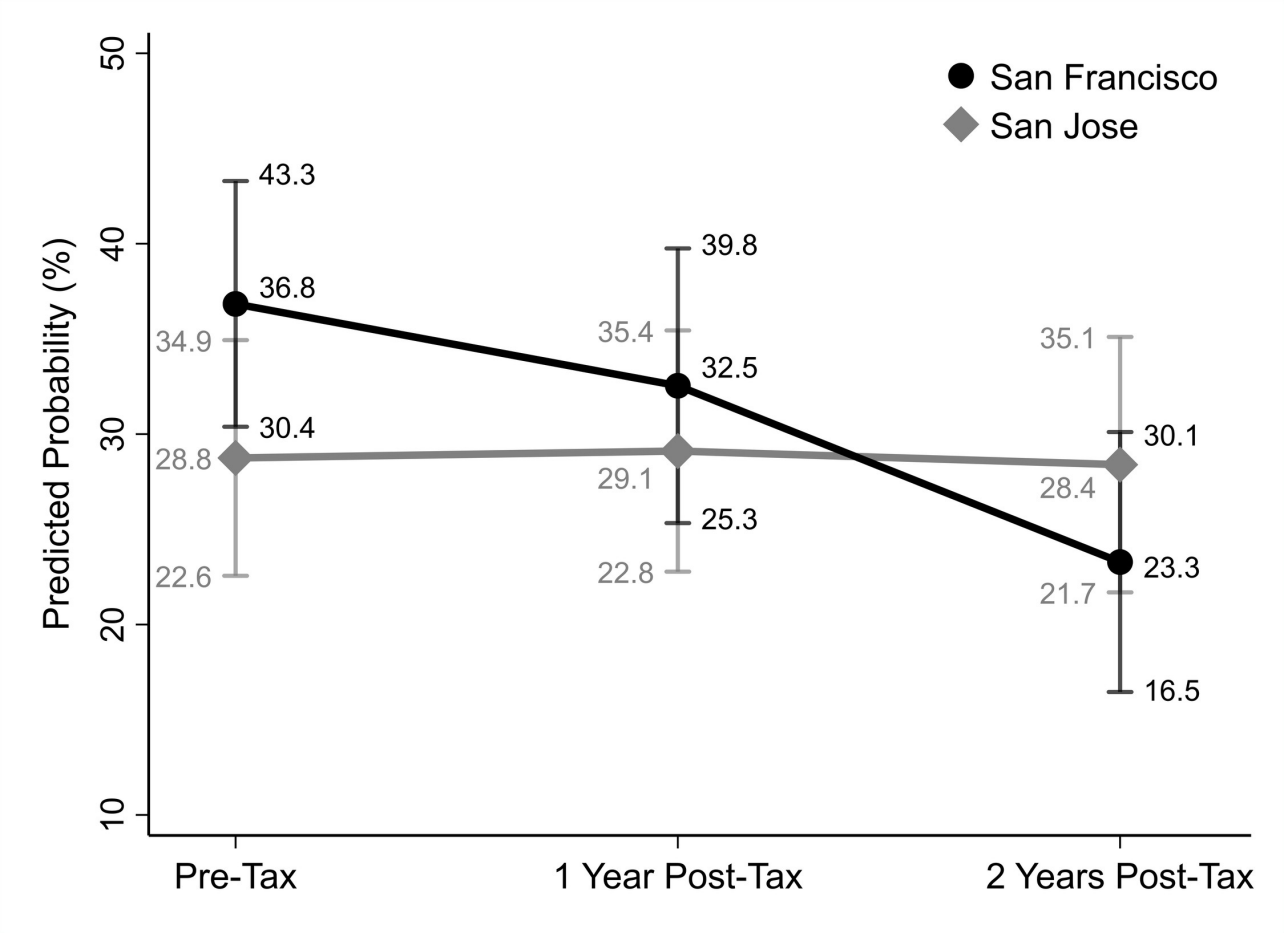
Average adjusted predicted probability of high SSB consumption^a^ with 95% confidence intervals in San Francisco and San Jose before, one, and two years after San Francisco’s sugar sweetened beverages tax implementation (n = 1,443). Note: Results based on binomial GLM with 2-way interactions between time (pre-tax, 1-year post-tax, and 2 years post-tax) and city (San Francisco, San José), controlling for age, sex, race/ethnicity, education, federal poverty level and sampling source. ^a^High SSB consumption refers to daily SSB consumption of ≥ 6 ounces per day.

At baseline, based on the unadjusted model, 37.3% (95% CI: 29.6, 44.9) of San Francisco respondents and 28.7% (95% CI: 22.3, 35.2) of San José respondents were high SSB consumers (≥ 6 ounces/178 ml daily); one-year post-tax, 33.0% (95% CI: 25.6, 40.4) of San Francisco and 29.0% (95% CI: 22.3, 35.7) of San José respondents were high consumers; and two-years post-tax 22.7% (95% CI:16.0, 29.5) of San Francisco and 28.2% (95% CI: 21.3, 35.1) of San José respondents were high consumers. The overall unadjusted two year 14.5% decline for San Francisco residents and 1% decline in San José were significantly different. In the adjusted binomial GLM, between the baseline and 1-year post-tax, there were no significant changes in odds of high SSB consumption in either San Francisco or San José (AOR[main effect] = 1.52, p = 0.080; AOR[interaction] = 0.79, p = 0.440). After 2 years, the odds of high intake decreased significantly for San Francisco residents and significantly more for San Francisco than for San José residents (AOR[interaction] = 0.49, p = 0.031; Table 3). Average adjusted predicted probabilities of high intake with 95% CIs pre- and post-tax are displayed in Fig 2. In San Francisco, the probability of consuming greater than 6 ounces (178 ml) per day decreased by 4.3% pre-tax to 1-year post-tax and decreased by 13.6% at 2 years post-tax. In San José, the probability of consuming 6 or more ounces (178 ml) per day increased less than 1% by 1-year post-tax and decreased by less than 1% at 2 years post-tax, a difference in change over time of 13.2% between cities. In sensitivity analyses examining high consumption using alternative thresholds for “high”, and in those excluding respondents from the first month of implementation, the adjusted binomial GLM’s for Year 2 yielded AOR’s for the interactions that were similar in magnitude and direction to those for the analysis using the 75^th^ percentile threshold; however, significance was not maintained in several models (Tables D and E in S1 Text).

**Table 3:** Difference-in-differences of likelihood of high sugar-sweetened beverage (SSB) consumption^a^ pre- and post-tax implementation between San Francisco and San José (n = 1,443).

	Unadjusted Odds Ratio (95% CI)	Adjusted Odds Ratio (95% CI)
**Time**		
Pre–Tax		1
Year 1 post–tax	1.01 (0.67,1.53)	1.02 (0.64,1.63)
Year 2 post–tax	0.97 (0.65,1.45)	0.98 (0.63,1.53)
**City**		
San Francisco	1.47 (0.93,2.32)	1.52 (0.95,2.43)
San José		1
**Difference-in-differences**		
City * Year 1 post–tax interaction	0.82 (0.48,1.40)	0.79 (0.43,1.44)
City * Year 2 post–tax interaction	0.51* (0.28,0.93)	0.49* (0.25,0.94)
Covariates		
Age category, years		0.98*** (0.97,0.99)
**Gender**		
Male		1
Female		0.75 (0.53,1.05)
**Race/Ethnicity**		
Asian or White		1
Black, Latino, or other race		1.63** (1.13,2.37)
**Education**		
Some college or lower		1.72** (1.24,2.39)
Bachelor’s degree or more		1
Federal Poverty Level		
Less than 200%		1.50* (1.03,2.17)
200% or greater		1
**Sampling Source**		
Random digit dialing		0.47*** (0.31,0.71)
Address–based		0.33*** (0.22,0.49)
Non–probability		1
**Constant**	0.40***(0.29,0.55)	1.15 (0.58,2.28)

Note

* = p < 0.05

** = p < 0.01

*** = p < 0.001; 1 = reference group; CI = confidence interval.

^a^High SSB consumption refers to 6 or more ounces of SSBs per day.

### Difference-in-differences across socioeconomic status and race/ethnicity GLM analyses 3

In the main binomial GLM analysis, we found that the odds of high intake decreased significantly for San Francisco residents only. The results of the 3-way-interaction model between federal poverty level (less than 200% versus greater than or equal to 200%), city, and time suggest that this difference-in-differences between cities pre- to 2 years post-tax is far greater among those living under 200% of the federal poverty level than those living at 200% or above (AOR[interaction] = 0.12, p = 0.010; Table F in S1 Text). The predicted probabilities of this model are presented in Table 4. Among those living under 200% of the federal poverty level, the average predicted probability of high SSB consumption decreased 23.6% in San Francisco and increased 17.7% in San José, yielding a difference-in-differences of 41.2%. Among those living at 200% or greater of the federal poverty level, there was less than 1 percentage point difference-in-differences.

**Table 4:** Average adjusted predicted probability of high SSB consumption^a^ in San Francisco and San Jose before, one, and two years after San Francisco’s sugar sweetened beverages tax implementation, stratified by federal poverty level (FPL) (n = 1,443).

	Pre-tax	1 Year post-tax	2 Years post-tax
Income as % Federal Poverty Level	Average probability estimate (95% CI)		
Less than 200%			
San Francisco	45.29 (31.81,58.78)	39.71 (25.08,54.34)	21.73 (10.29,33.18)
San José	28.19 (15.19,41.20)	29.77 (18.57,40.97)	45.86 (28.47,63.24)
200% or greater			
San Francisco	32.35 (25.44,39.26)	28.64 (21.00,36.28)	24.32 (15.94,32.70)
San Jose	28.55 (21.60,35.49)	28.69 (21.08,36.31)	21.17 (14.61,27.73)

Note: CI = confidence interval. Results based on binomial GLM with 3-way interactions between time (pre-tax, 1-year post-tax, and 2 years post-tax), city (San Francisco, San Jose), and federal poverty level, controlling for age, sex, race/ethnicity, education, and sampling source.

^a^High SSB consumption refers to 6 or more ounces of SSBs per day.

The three-way interaction binomial models examining probability of high SSB consumption for education and race/ethnicity had non-significant interactions (not shown).

In addition, for the continuous outcome of SSB consumption measured in ounces, the Gamma GLMs examining change over time with 3-way interactions between demographic (federal poverty level, race/ethnicity, and education), year, and city were non-significant, suggesting that the difference-in-differences of overall SSB consumption between cities did not vary significantly by socioeconomic status or race/ethnicity (not shown). We found no evidence that the difference-in-differences in the Gamma and binomial models varied by sample source (not shown).

### Sensitivity analyses

These sensitivity analyses exchanged city of residence with the dichotomized days (<16 versus ≥ 16 days) spent in San Francisco in the past 30 days as the measure of exposure to SSB taxes in the main Gamma and binomial GLM’s. In the analyses, 505 of the 602 San Francisco residents spent 16 days or more days in San Francisco compared to 5 of the 710 San José residents. The sensitivity analyses produced findings consistent with both SSB consumption (ounces) and high SSB consumption models (Tables G and H in S1 Text).

### Cross border shopping

San Francisco residents were significantly more likely to report changing to buy more at locations outside their city compared to their San José counterparts at every time point (4.4% vs 1.3% at baseline; 6.1% vs. 0.9% at F1; and 3.9% vs 2.0% at F2). However, the difference-in-differences of the adjusted binomial GLM over time was not significant from pre-tax to year 1 (AOR[interaction] = 2.11, p = 0.387) or year 2 post-tax (AOR[interaction] = 0.55, p = 0.511), suggesting the SSB tax did not significantly affect rate of change in cross-border shopping practices (Table I in S1 Text).

## Discussion

The difference-in-differences findings at two years in the studied areas are supportive of the effectiveness of a one cent per ounce tax on SSBs in reducing high SSB consumption. Average SSB intake fell significantly in San Francisco at one and two years post tax, but difference in differences between cities was not significant. Our findings provide stronger evidence of effectiveness in reduction of SSB consumption, though limited to high consumption, than the findings of Cawley and Frisvold who did not find change in the combined Oakland, San Francisco and Seattle data in the first six months post tax [22]. Findings of greater reductions in lower-income consumers are consistent with greater sensitivity to price changes among this population and with tax evaluation findings from Mexico, Thailand and Catalunya [29,46,47]. In contrast, in Chile, with high sugary beverages (≥6.25g/100ml) taxed 8% more than less sugary beverages, both Nakamura and Caro found decreases in SSB purchase volumes (of -21.6% and—3.4%, respectively) that were greater in higher socioeconomic groups [48,49].

Our findings suggest that the likelihood of being a “high” SSB consumer, even at the level of six ounces (178 ml) per day, declined significantly more in these San Francisco neighborhoods than in those of San José between the baseline and the end of the second year of the tax. Of note, in these relatively low SSB consuming cities, the top quartile of SSB consumption was above 6 ounces, similar to Sánchez-Romero (≥one serving per day) [26] but lower than the highest mortality quartile of Anderson’s United Kingdom study assessing SSB consumption and mortality risk (>2 servings a day) [4]. Here, the difference-in-differences was more pronounced amongst those living in poverty, below 200% of the federal poverty level, compared with those living at least 200% above the federal poverty level, consistent with findings from Mexico [46]. Differences by city over time did not significantly vary based on race/ethnicity or education, although those factors were significantly associated with likelihood of being a high SSB consumer. This study did not examine the role of mediating factors such as attitudes towards SSBs and perception of harm, which may also be factors influencing behavior change. There was no significant difference-in-differences in change to cross-border shopping reported, a factor which can influence both tax impact on SSB intake and tax revenue for taxes limited to a city. Cross-border shopping may have been constrained by both San Francisco’s water-bound geography and the fact that three nearby cities also tax SSBs. While this study was limited to the outcome of self-reported SSB consumption, further research using different conceptual approaches and assessment of longer-term health outcomes are needed to fully assess these policies.

### Strengths

Strengths of the study include the longer time frame of two years post-tax, the use of individual consumption data, the large sample size, the assessment of potential moderating factors, and oversampling of Blacks and Latinos who are typically higher consuming racial and ethnic groups that constitute a small but important part of the intervention and control city populations. Use of individual consumption data may also help capture the impact of the tax in restaurant and other settings not reflected in studies based on electronic sales data.

### Limitations

Nevertheless, certain limitations should be noted. First, because the sample was primarily drawn from neighborhoods with greater density of Black and Latino residents, it is therefore not representative of the two cities as a whole. Second, the control city, San José, is close to the intervention site (64 km) and other nearby Bay Area cities with newly adopted tax policies, sharing certain media markets, distribution systems and public health practices. This could potentially lead to underestimation of differences in tax effects if there was regional pricing of beverages by chain retailers, for example. San José may also have shared the “risk signaling” effect of the Bay Area SSB tax measures [50]. Cawley and Frisvold found greater difference-in-differences using a broader synthetic national control than controls in areas adjacent to taxing cities [22]. Similarly, if social norms around beverage consumption in the region underwent broader shifts because of the taxes, it could reduce the difference-in-differences. Other policies were also put into place during an overlapping time period, including elimination of SSB sales at a major employer and on city property in San Francisco, and educational campaigns continued in both cities. In general, there has been a secular trend in California towards reduced consumption of SSBs between 2011 and 2018 in all age groups, and a 10% decline in calories nationally from non-alcoholic beverages between 2014 and 2020 [51,52]. This may be a result of taxation policies, threat of taxation, education, or other SSB policy interventions and consequent shifts in product portfolios. Thus, results may be conservative. Baseline data collection extended 23 days into the initial implementation period of the tax to collect sufficient respondents, which could also lead to underestimation of effect, although sensitivity analyses does not suggest that to be the case.

Recruitment challenges for this time sensitive work necessitated a complex sampling design. For these reasons we applied weighting to account for the various sample sources and focused solely on change over time in those respondents who were captured on follow-up at one or more time points. While baseline participants from the non-probability sample were more likely to drop out, sampling source-based attrition rates did not differ by city. We also found no evidence of difference-in-differences findings varying by sampling source. We accounted for the differential attrition by predicting the probability of continuing participation at each wave by city, demographic characteristics and their SSB consumption and then inversely weighting the estimated probabilities before the post-stratification. Despite this, we recognize there may be still concern that the final sample is less representative of our population of interest because of this type of attrition. Consumption is based on self-reported estimates of the preceding month’s consumption, and though other U.S. data have shown month-to-month SSB consumption to be relatively stable [53], monthly recall may be less reliable than 24-hour dietary intake. The lack of statistical significance in the overall consumption outcome despite reasonable sample size also highlights the difficulties in using self-reported consumption and survey research to assess these policies. With high cost and declining response rates [36,54], using telephone or mail survey methods to detect significant changes over time and between locations can be challenging for a behavior with high variance. Use of sales-based electronic data may offer greater power and a more reliable picture of SSB-related changes in a geographic area but is limited in its ability to permit sub-group or individual-level analyses and may provide less complete capture of sites of purchase or consumption. It is also notable that these are two cities with far lower proportions of racial and ethnic groups with high SSB consumption than Philadelphia, which may account for variation in policy impact across cities. This study did not examine the justice of the economic burden or benefits of the tax, but the significant intake reduction in low-income high consumers suggests potential benefit for those at highest risk for health harms.

Finally, this study reflected a relatively modest size tax, limited to sugar sweetened beverages above 25 kcal per 12 ounces, and not tiered for sugar content, and therefore cannot be generalized to the wide variety of taxation models in use globally. Notably, the World Health Organization has recommended SSB taxes of 20% or greater to prevent noncommunicable disease [55].

### Policy implications

This study contributes to the growing body of literature assessing impact of sugar sweetened beverage tax policies. It suggests that even a modest size tax can be effective in reducing high consumption, especially for those of lower income, and that reduction grows over two years, but that higher taxation and/or additional taxation strategies may be necessary to significantly reduce population wide SSB consumption patterns.

## Conclusion

In summary, two years after implementation, the modest San Francisco SSB tax appears to be associated with significantly greater reductions in the likelihood of being a high SSB consumer in the studied neighborhoods, especially amongst those living in poverty. A -34.1% change in overall consumption of SSBs in ounces in San Francisco *versus* a -16.5% change in San José by year two was a non-significant difference-in-differences. Longer periods of study may be valuable for assessing tax effects.

## Supporting information

S1 Text **Fig A.** Sampling Method Flowchart. **Table A.** Characteristics of sample at baseline, by sampling source (2017–2018) (n = 1,433). **Table B.** Characteristics of sample at baseline, by city and survey date (2017–2018) (n = 1,433). **Table C.** Difference-in-differences of sugar-sweetened beverage consumption (ounces) pre- and post-tax implementation between San Francisco and San José, among participants who took baseline survey prior to January 1^st^, 2018 (n = 980). **Table D.** Difference-in-differences of likelihood of high sugar-sweetened beverage (SSB) consumption pre- and post-tax implementation between San Francisco and San José, using varying thresholds for high versus low consumption (n = 1,433). **Table E.** Difference-in-differences of likelihood of high sugar-sweetened beverage (SSB) consumption pre- and post-tax implementation between San Francisco and San José, among participants who took baseline survey prior to January 1^st^, 2018 (n = 980). **Table F.** Generalized linear model of high sugar-sweetened beverage consumption in San Francisco and San Jose before, one, and two years after San Francisco’s sugar sweetened beverages tax implementation, with 3-way interactions between city, year, and federal poverty level (FPL) (n = 1,433). **Table G.** Difference-in-differences in sugar-sweetened beverage consumption (ounces) pre- and post- SSB tax implementation between adults who spent 16 or more *versus* fewer days in San Francisco (n = 1,312). **Table H.** Difference-in-differences of high vs low sugar-sweetened beverage consumption^a^ pre- and post- SSB tax implementation between adults who spent 16 or more *versus* fewer days in San Francisco (n = 1,312). **Table I.** Difference-in-differences of likelihood of changing city of purchase for sugar-sweetened beverages (SSB) pre- and post-tax implementation between San Francisco and San José (n = 1,443).
(DOCX)
