# Correction: Changes in sugar-sweetened beverage consumption in the first two years (2018–2020) of San Francisco’s tax: A prospective longitudinal study

**DOI:** 10.1371/journal.pgph.0004820

**Published:** 2025-06-17

**Authors:** Lynn D. Silver, Alisa A. Padon, Libo Li, Bethany J. Simard, Thomas K. Greenfield

The Data Availability statement for this article is incorrect. The correct statement is: Silver LD, Padon AA, Li L, Simard B, Greenfield TK. Changes in sugar-sweetened beverage consumption in the first two years (2018–2020) of San Francisco’s tax: A prospective longitudinal study 2022. Dryad Digital Repository. https://doi.org/10.5061/dryad.hhmgqnkkq.
