# Supplementary material for: Changes in sugar-sweetened beverage consumption in the first two years (2018 – 2020) of San Francisco’s tax: A prospective longitudinal study
Source: PLOS Glob Public Health. 2023 Jan 25;3(1):e0001219. doi: 10.1371/journal.pgph.0001219 (PMC10021346; doi:10.1371/journal.pgph.0001219)
Supplement: S1 Text — Fig A. Sampling Method Flowchart. Table A. Characteristics of sample at baseline, by sampling source (2017–2018) (n = 1,433). Table B. Characteristics of sample at baseline, by city and survey date (2017–2018) (n = 1,433). Table C. Difference-in-differences of sugar-sweetened beverage consumption (ounces) pre- and post-tax implementation between San Francisco and San José, among participants who took baseline survey prior to January 1st, 2018 (n = 980). Table D. Difference-in-differences of likelihood of high sugar-sweetened beverage (SSB) consumption pre- and post-tax implementation between San Francisco and San José, using varying thresholds for high versus low consumption (n = 1,433). Table E. Difference-in-differences of likelihood of high sugar-sweetened beverage (SSB) consumption pre- and post-tax implementation between San Francisco and San José, among participants who took baseline survey prior to January 1st, 2018 (n = 980). Table F. Generalized linear model of high sugar-sweetened beverage consumption in San Francisco and San Jose before, one, and two years after San Francisco’s sugar sweetened beverages tax implementation, with 3-way interactions between city, year, and federal poverty level (FPL) (n = 1,433). Table G. Difference-in-differences in sugar-sweetened beverage consumption (ounces) pre- and post- SSB tax implementation between adults who spent 16 or more versus fewer days in San Francisco (n = 1,312). Table H. Difference-in-differences of high vs low sugar-sweetened beverage consumptiona pre- and post- SSB tax implementation between adults who spent 16 or more versus fewer days in San Francisco (n = 1,312). Table I. Difference-in-differences of likelihood of changing city of purchase for sugar-sweetened beverages (SSB) pre- and post-tax implementation between San Francisco and San José (n = 1,443). (DOCX) [file pgph.0001219.s001.docx]

**Supporting Information**

**Changes in sugar-sweetened beverage consumption in the first two years (2018 – 2020) of San Francisco’s tax: A prospective longitudinal study**

**Fig A.** Sampling Method Flowchart

**Table A.** Characteristics of sample at baseline, by sampling source (2017–2018) (n=1,433)

**Table B.** Characteristics of sample at baseline, by city and survey date (2017–2018) (n=1,433)

**Table C.** Difference-in-differences of sugar-sweetened beverage consumption (ounces) pre- and post-tax implementation between San Francisco and San José, among participants who took baseline survey prior to January 1^st^, 2018 (n=980)

**Table D.** Difference-in-differences of likelihood of high sugar-sweetened beverage (SSB) consumption pre- and post-tax implementation between San Francisco and San José, using varying thresholds for high versus low consumption (n=1,433)

**Table E.** Difference-in-differences of likelihood of high sugar-sweetened beverage (SSB) consumption pre- and post-tax implementation between San Francisco and San José, among participants who took baseline survey prior to January 1^st^, 2018 (n=980)

**Table F.** Generalized linear model of high sugar-sweetened beverage consumption in San Francisco and San Jose before, one, and two years after San Francisco’s sugar sweetened beverages tax implementation, with 3-way interactions between city, year, and federal poverty level (FPL) (n=1,433)

**Table G.** Difference-in-differences in sugar-sweetened beverage consumption (ounces) pre- and post- SSB tax implementation between adults who spent 16 or more *versus* fewer days in San Francisco (n=1,312)

**Table H.** Difference-in-differences of high vs low sugar-sweetened beverage consumption^a^ pre- and post- SSB tax implementation between adults who spent 16 or more *versus* fewer days in San Francisco (n=1,312)

**Table I.** Difference-in-differences of likelihood of changing city of purchase for sugar-sweetened beverages (SSB) pre- and post-tax implementation between San Francisco and San José (n=1,443)

**Fig A. Sampling Method Flowchart**

**
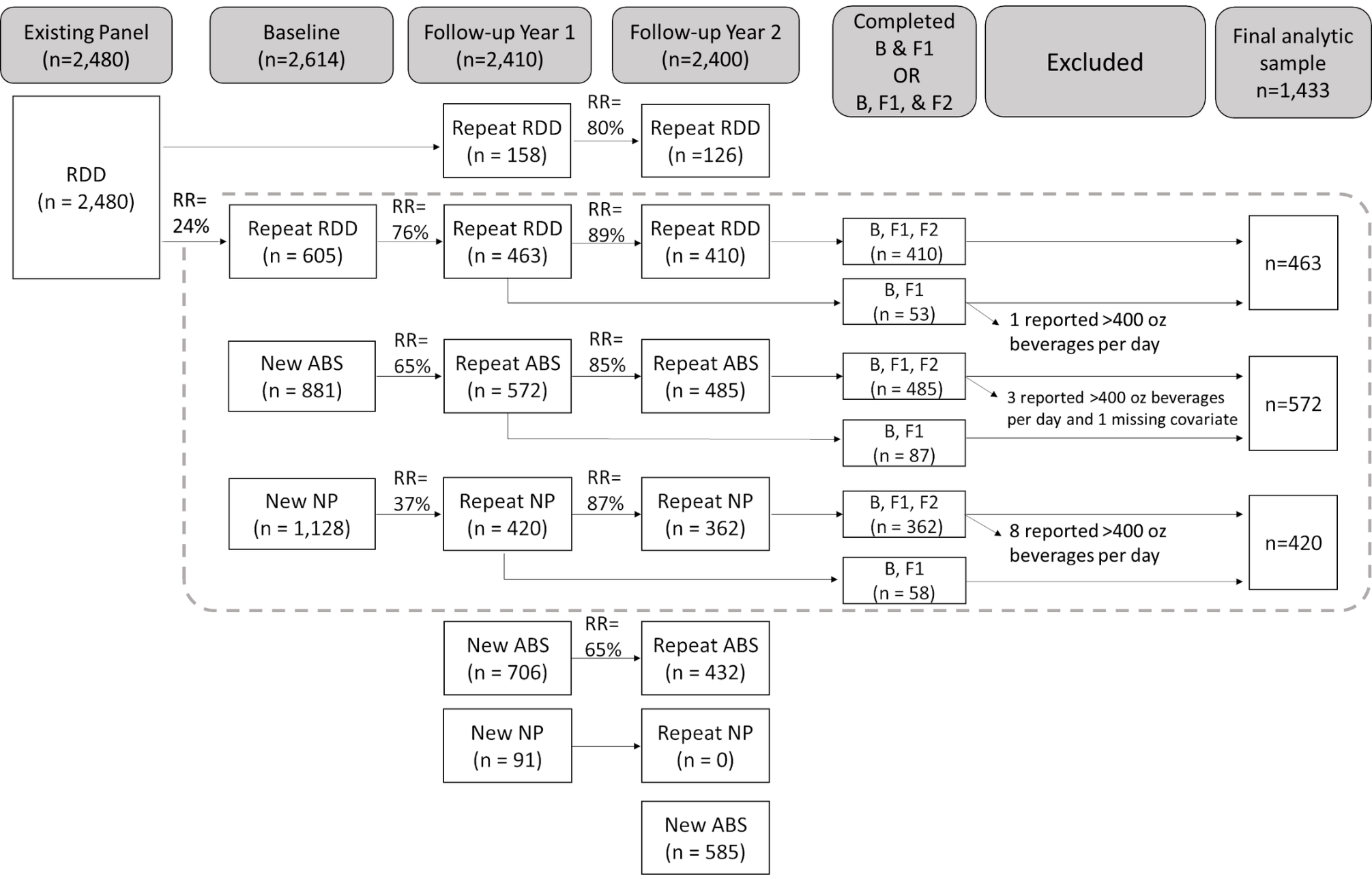
**

Note: RDD=Random Digit-dial; ABS = Address-based Sampling; NP=Non-probability sample; RR = Return Rate

**Table A. Characteristics of sample at baseline, by sampling source (2017–2018) (n=1,433)**

|  | Random-Digit Dial | Address-Based | Non-probability | p–Value^a^ |
| --- | --- | --- | --- | --- |
|  | (n = 458) | (n = 569) | (n = 412) |  |
| Age, years, weighted median (IQR) | 38 (29–55) | 42 (29–55) | 39 (29–56) | <0.001 |
|  | *n (weighted %)* | | |  |
| Sex |  |  |  |  |
| Male | 197 (54.1) | 269 (50.6) | 188 (48.7) | 0.698 |
| Female | 265 (45.9) | 300 (49.4) | 224 (51.3) |  |
| Race/Ethnicity |  |  |  |  |
| White | 279 (32.7) | 295 (33.6) | 229 (33.2) | 0.887 |
| Asian | 66 (27.4) | 158 (31.5) | 93 (32.1) |  |
| Latino | 63 (26.7) | 77 (26.4) | 62 (26.2) |  |
| Black | 30 (6.0) | 22 (4.3) | 20 (5.5) |  |
| Other | 24 (7.2) | 17 (4.1) | 8 (3.0) |  |
| Education |  |  |  |  |
| High School or Less | 28 (33.6) | 44 (39.4) | 36 (39.7) | 0.770 |
| Some College | 98 (27.2) | 110 (24.4) | 106 (22.3) |  |
| 4–year College | 173 (24.0) | 214 (23.6) | 164 (25.6) |  |
| Graduate or Professional School | 163 (15.2) | 201 (12.6) | 106 (12.4) |  |
| Federal Poverty Level |  |  |  |  |
| Less than 200% | 79 (28.7) | 92 (34.8) | 72 (29.8) | 0.574 |
| 200% or greater | 383 (71.3) | 477 (65.2) | 340 (70.2) |  |

Note: SSB = sugar sweetened beverage; IQR = interquartile range. “Other race” includes participants who identified as Native Hawaiian or Pacific Islander, American Indian or Alaska Native, or some other race.

^a^p–value of Chi–square test for differences in covariates across cities for all characteristics except age and average daily consumption measured in ounces, for which Mood’s median test for differences in medians across cities was conducted.

**Table B. Characteristics of sample at baseline, by city and survey date (2017–2018) (n=1,433)**

|  | San Francisco | | | San José | | |
| --- | --- | --- | --- | --- | --- | --- |
|  | Baseline <1/2018  (n=565) | Baseline 1/2018  (n=157) | p–Value^a^ | Baseline <1/2018  (n=415) | Baseline 1/2018  (n=306) | p–Value^a^ |
|  |  |  |  |  |  |  |
| Age, years, weighted median (IQR) | 38 (27–54) | 46 (38–60) | p<0.001 | 37 (30-49) | 44 (29–57) | p<0.001 |
|  | *n (weighted %)* | | | | | |
| Sex |  |  |  |  |  |  |
| Male | 256 (52.8) | 69 (49.8) | 0.720 | 180 (51.2) | 149 (49.9) | 0.850 |
| Female | 309 (47.2) | 88 (50.2) |  | 235 (48.8) | 157 (50.1) |  |
| Race/Ethnicity |  |  |  |  |  |  |
| White | 322 (30.0) | 88 (30.7) | 0.189 | 227 (32.4) | 166 (39.1) | 0.672 |
| Asian | 123 (31.3) | 40 (45.0) |  | 82 (26.8) | 72 (28.8) |  |
| Latino | 60 (24.3) | 15 (18.0) |  | 82 (31.7) | 45 (24.8) |  |
| Black | 43 (12.2) | 12 (5.2) |  | 9 (1.5) | 8 (1.6) |  |
| Other | 17 (2.1) | 2 (1.2) |  | 15 (7.6) | 15 (5.7) |  |
| Education |  |  |  |  |  |  |
| High School or Less | 43 (39.8) | 10 (30.3) | 0.527 | 33 (40.0) | 22 (33.8) | 0.585 |
| Some College | 117 (22.1) | 21 (22.9) |  | 106 (25.0) | 70 (28.3) |  |
| 4–year College | 225 (25.0) | 65 (32.1) |  | 136 (20.9) | 125 (25.3) |  |
| Graduate or Professional School | 180 (13.1) | 61 (14.7) |  | 140 (14.0) | 89 (12.6) |  |
| Federal Poverty Level |  |  |  |  |  |  |
| Less than 200% | 113 (37.9) | 27 (37.2) | 0.938 | 57 (24.7) | 46 (30.5) | 0.394 |
| 200% or greater | 452 (62.1) | 130 (62.8) |  | 358 (75.3) | 260 (69.5) |  |
| Sampling Source |  |  |  |  |  |  |
| Random digit dialing | 185 (31.9) | 46 (28.3) | <0.001 | 176 (43.3) | 55 (24.6) | 0.007 |
| Address–based | 152 (29.5) | 110 (70.0) |  | 134 (30.5) | 173 (51.4) |  |
| Non–probability | 228 (38.6) | 1 (1.7) |  | 105 (26.2) | 78 (24.0) |  |

Note: IQR = interquartile range. “Other race” includes participants who identified as Native Hawaiian or Pacific Islander, American Indian or Alaska Native, or some other race.

^a^p–value of Chi–square test for differences in covariates across cities for all characteristics except age and average daily consumption measured in ounces, for which Mood’s median test for differences in medians across cities was conducted.

**Table C. Difference-in-differences of sugar-sweetened beverage consumption (ounces) pre- and post-tax implementation between San Francisco and San José, among participants who took baseline survey prior to January 1^st^, 2018 (n=980)**

|  | Adjusted  Arithmetic Mean Ratio^a^  (95% CI) |
| --- | --- |
|  |  |
| **Time** |  |
| Pre–Tax | 1 |
| Year 1 post–tax | 0.79 (0.58,1.06) |
| Year 2 post–tax | 0.94 (0.71,1.25) |
| **City** |  |
| San Francisco | 1.75** (1.23,2.50) |
| San José |  |
| **Difference-in-differences** |  |
| City * Year 1 post–tax interaction | 0.86 (0.58,1.27) |
| City * Year 2 post–tax interaction | 0.75 (0.48,1.16) |
| Covariates |  |
| Age, years | 0.98*** (0.97,0.99) |
| Sex |  |
| Male | 1 |
| Female | 0.81 (0.58,1.13) |
| Race/Ethnicity |  |
| Asian or White | 1 |
| Black, Latino, or other race | 1.42 (0.99,2.04) |
| Education |  |
| Some college or lower | 1.41 (0.99,2.02) |
| Bachelor’s degree or more | 1 |
| Federal Poverty Level |  |
| Less than 200% | 1.74** (1.24,2.43) |
| 200% or greater | 1 |
| Sampling Source |  |
| Random digit dialing | 0.46*** (0.32,0.68) |
| Address–based | 0.45*** (0.30,0.67) |
| Non–probability | 1 |
| Constant | 14.81*** (8.58,25.58) |

Note: * = p < 0.05, ** = p < 0.01, *** = p < 0.001; 1 = reference group; CI = confidence interval.

^a^Exponentiated coefficients of Gamma GLM of SSB consumption in ounces.

**Table D. Difference-in-differences of likelihood of high sugar-sweetened beverage (SSB) consumption pre- and post-tax implementation between San Francisco and San José, using varying thresholds for high versus low consumption (n=1,433)**

|  | **High SSB consumption threshold** | | | |
| --- | --- | --- | --- | --- |
|  | **≥4 ounces** | **≥8 ounces** | **≥12 ounces** | |
|  | **Adjusted Odds Ratio (95% CI)** | | | |
|  |  |  | |  |
| **Time** |  |  | |  |
| Pre–Tax | 1 | 1 | | 1 |
| Year 1 post–tax | 1.04 (0.71,1.51) | 0.72 (0.46,1.14) | | 0.85 (0.54,1.33) |
| Year 2 post–tax | 0.85 (0.57,1.27) | 0.85 (0.52,1.40) | | 0.72 (0.40,1.30) |
| **City** |  |  | |  |
| San Francisco | 1.49 (0.97,2.29) | 1.33 (0.81,2.21) | | 1.52 (0.86,2.68) |
| San José | 1 | 1 | | 1 |
| **Difference-in-differences** |  |  | |  |
| City * Year 1 post–tax interaction | 0.66 (0.39,1.13) | 1.09 (0.58,2.07) | | 0.84 (0.44,1.61) |
| City * Year 2 post–tax interaction | 0.64 (0.38,1.08) | 0.60 (0.30,1.19) | | 0.80 (0.37,1.75) |
| Covariates |  |  | |  |
| Age, years | 0.97*** (0.96,0.98) | 0.98** (0.97,0.99) | | 0.98** (0.97,0.99) |
| **Gender** |  |  | |  |
| Male | 1 | 1 | | 1 |
| Female | 0.68* (0.47,0.98) | 0.86 (0.60,1.25) | | 0.85 (0.55,1.31) |
| **Race/Ethnicity** |  |  | |  |
| Asian or White | 1 | 1 | | 1 |
| Black, Latino, or other race | 1.54* (1.03,2.30) | 1.77** (1.16,2.70) | | 1.76* (1.08,2.86) |
| **Education** |  |  | |  |
| Some college or lower | 1.87*** (1.33,2.63) | 1.75** (1.23,2.50) | | 2.10*** (1.42,3.12) |
| Bachelor’s degree or more | 1 | 1 | | 1 |
| **Federal Poverty Level** |  |  | |  |
| Less than 200% | 1.27 (0.87,1.87) | 1.43 (0.95,2.14) | | 1.29 (0.81,2.05) |
| 200% or greater | 1 | 1 | | 1 |
| **Sampling Source** |  |  | |  |
| Random digit dialing | 0.40*** (0.26,0.62) | 0.51** (0.32,0.82) | | 0.55* (0.31,0.96) |
| Address–based | 0.35*** (0.23,0.53) | 0.37*** (0.24,0.56) | | 0.42*** (0.26,0.67) |
| Non–probability | 1 | 1 | | 1 |
| **Constant** | 2.33* (1.19,4.54) | 0.71 (0.34,1.45) | | 0.41* (0.18,0.97) |

Note: * = p < 0.05, ** = p < 0.01, *** = p < 0.001; 1 = reference group; CI = confidence interval.

**Table E. Difference-in-differences of likelihood of high sugar-sweetened beverage (SSB) consumption pre- and post-tax implementation between San Francisco and San José, among participants who took baseline survey prior to January 1^st^, 2018 (n=980)**

|  | **High SSB consumption threshold** | | | |
| --- | --- | --- | --- | --- |
|  | **≥4 ounces** | **≥6 ounces^a^** | **≥8 ounces** | **≥12 ounces** |
|  | **Adjusted Odds Ratio (95% CI)** | | | |
|  |  |  |  |  |
| **Time** |  |  |  |  |
| Pre–Tax | 1 | 1 | 1 | 1 |
| Year 1 post–tax | 1.03 (0.69,1.54) | 0.92 (0.54,1.56) | 0.62 (0.35,1.11) | 0.65 (0.37,1.16) |
| Year 2 post–tax | 0.75 (0.49,1.14) | 0.87 (0.54,1.38) | 0.78 (0.44,1.37) | 0.67 (0.36,1.27) |
| **City** |  |  |  |  |
| San Francisco | 1.71* (1.02,2.87) | 1.97* (1.14,3.42) | 1.62 (0.89,2.93) | 1.81 (0.94,3.47) |
| San José | 1 | 1 | 1 | 1 |
| **Difference-in-differences** |  |  |  |  |
| City * Year 1 post–tax interaction | 0.65 (0.36,1.20) | 0.79 (0.39,1.60) | 1.20 (0.52,2.77) | 0.90 (0.39,2.06) |
| City * Year 2 post–tax interaction | 0.72 (0.41,1.27) | 0.44* (0.22,0.86) | 0.54 (0.26,1.14) | 0.75 (0.33,1.69) |
| Covariates |  |  |  |  |
| Age, years | 0.97*** (0.96,0.98) | 0.97*** (0.96,0.98) | 0.97*** (0.96,0.99) | 0.97*** (0.95,0.98) |
| **Gender** |  |  |  |  |
| Male | 1 | 1 | 1 | 1 |
| Female | 0.77 (0.49,1.22) | 0.89 (0.58,1.37) | 1.03 (0.66,1.61) | 1.19 (0.70,2.01) |
| **Race/Ethnicity** |  |  |  |  |
| Asian or White | 1 | 1 | 1 | 1 |
| Black, Latino, or other race | 1.22 (0.74,2.03) | 1.39 (0.86,2.26) | 1.42 (0.86,2.35) | 1.65 (0.93,2.94) |
| **Education** |  |  |  |  |
| Some college or lower | 1.86** (1.23,2.83) | 1.61* (1.09,2.37) | 1.67* (1.09,2.55) | 1.68* (1.04,2.69) |
| Bachelor’s degree or more | 1 | 1 | 1 | 1 |
| **Federal Poverty Level** |  |  |  |  |
| Less than 200% | 1.40 (0.84,2.35) | 1.48 (0.90,2.42) | 1.40 (0.86,2.29) | 1.22 (0.70,2.13) |
| 200% or greater | 1 | 1 | 1 | 1 |
| **Sampling Source** |  |  |  |  |
| Random digit dialing | 0.38*** (0.23,0.63) | 0.44*** (0.28,0.71) | 0.54* (0.32,0.92) | 0.51* (0.27,0.94) |
| Address–based | 0.35*** (0.19,0.62) | 0.30*** (0.17,0.53) | 0.37** (0.21,0.68) | 0.43** (0.22,0.81) |
| Non–probability | 1 | 1 | 1 | 1 |
| **Constant** | 2.62* (1.17,5.86) | 1.44 (0.62,3.38) | 0.88 (0.38,2.07) | 0.62 (0.22,1.73) |

Note: * = p < 0.05, ** = p < 0.01, *** = p < 0.001; 1 = reference group; CI = confidence interval.

^a^Used in main analyses

**Table F. Generalized linear model of high sugar-sweetened beverage consumption in San Francisco and San Jose before, one, and two years after San Francisco’s sugar sweetened beverages tax implementation, with 3-way interactions between city, year, and federal poverty level (FPL) (n=1,433)**

|  | Adjusted Odds Ratio  (95% CI) |
| --- | --- |
|  |  |
| Time |  |
| Pre–Tax | 1 |
| Year 1 post–tax | 1.01 (0.59,1.72) |
| Year 2 post–tax | 0.65 (0.40,1.04) |
| City |  |
| San Francisco | 1.22 (0.74,2.01) |
| San Jose | 1 |
| Federal Poverty Level |  |
| Less than 200% | 0.98 (0.43,2.23) |
| Greater than or equal to 200% | 1 |
| Two-way Interactions |  |
| City * Year 1 post–tax | 0.82 (0.42,1.57) |
| City * Year 2 post–tax | 1.00 (0.51,1.95) |
| City * FPL[<200%] | 1.89 (0.66,5.44) |
| Year 1 post–tax * FPL[<200%] | 1.08 (0.38,3.05) |
| Year 2 post–tax * FPL[<200%] | 3.68^*^ (1.19,11.37) |
| Three-way Interactions |  |
| City * Year 1 post–tax * FPL[<200%] | 0.87 (0.23,3.29) |
| City * Year 2 post–tax * FPL[<200%] | 0.12^*^ (0.03,0.61) |
| Covariates |  |
| Age, years | 0.98^***^ (0.97,0.99) |
| Sex |  |
| Male | 1 |
| Female | 0.75 (0.54,1.05) |
| Race/Ethnicity |  |
| Asian or White | 1 |
| Black, Latino, or other race | 1.66^**^ (1.15,2.38) |
| Education |  |
| Some college or lower | 1.73^***^ (1.25,2.40) |
| Bachelor’s degree or more | 1 |
| Sampling Source |  |
| Random digit dialing | 0.47^***^ (0.32,0.70) |
| Address–based | 0.33^***^ (0.22,0.50) |
| Non–probability |  |
| Constant | 1.31 (0.66,2.61) |

Note: * = p < 0.05, ** = p < 0.01, *** = p < 0.001; 1 = reference group; CI = confidence interval.

**Table G. Difference-in-differences in sugar-sweetened beverage consumption (ounces) pre- and post- SSB tax implementation between adults who spent 16 or more *versus* fewer days in San Francisco (n=1,312)**

|  | Arithmetic Mean Ratio^a^  (95% CI) |
| --- | --- |
|  |  |
| Time |  |
| Pre–Tax | 1 |
| Year 1 post–tax | 0.90 (0.62,1.31) |
| Year 2 post–tax | 0.83 (0.65,1.07) |
| Days spent in San Francisco in past 30 days |  |
| Fewer than 16 days | 1 |
| 16 or more days | 1.50* (1.08,2.09) |
| Difference-in-differences |  |
| 16 or more days * Year 1 post–tax | 0.76 (0.48,1.19) |
| 16 or more days * Year 2 post–tax | 0.70 (0.45,1.07) |
| Covariates |  |
| Age category, years | 0.99** (0.98,1.00) |
| Sex |  |
| Male | 1 |
| Female | 0.69** (0.52,0.91) |
| Race/Ethnicity |  |
| Asian or White | 1 |
| Black, Latino, or other race | 1.51** (1.12,2.04) |
| Education |  |
| Some college or lower | 1.76*** (1.31,2.37) |
| Bachelor’s degree or more | 1 |
| Federal Poverty Level |  |
| Less than 100% | 1.30 (0.99,1.70) |
| 200% or greater | 1 |
| Sampling Source |  |
| Random digit dialing | 0.52*** (0.36,0.74) |
| Address–based | 0.43*** (0.32,0.58) |
| Non–probability | 1 |
| Constant | 12.20*** (7.36,20.22) |

Note: * = p < 0.05, ** = p < 0.01, *** = p < 0.001; 1 = reference group; CI = confidence interval.

**Table H. Difference-in-differences of high vs low sugar-sweetened beverage consumption^a^ pre- and post- SSB tax implementation between adults who spent 16 or more *versus* fewer days in San Francisco (n=1,312)**

|  | Adjusted Odds Ratio (95% CI) |
| --- | --- |
|  |  |
| Time |  |
| Pre–Tax | 1 |
| Year 1 post–tax | 0.95 (0.61,1.46) |
| Year 2 post–tax | 0.99 (0.64,1.52) |
| Days Spent in San Francisco in past 30 days |  |
| Fewer than 16 days | 1 |
| 16 or more days | 1.63 (0.99,2.70) |
| Difference-in-differences |  |
| 16 or more days * Year 1 post–tax | 0.82 (0.43,1.55) |
| 16 or more days * Year 2 post–tax | 0.43* (0.21,0.91) |
| Covariates |  |
| Age, years | 0.98*** (0.97,0.99) |
| Sex |  |
| Male | 1 |
| Female | 0.74 (0.51,1.06) |
| Race/Ethnicity |  |
| Asian or White | 1 |
| Black, Latino, or other race | 1.67* (1.13,2.47) |
| Education |  |
| Some college or lower | 1.60** (1.14,2.26) |
| Bachelor’s degree or more | 1 |
| Less than 200% |  |
| Less than 200% | 1.46 (0.98,2.16) |
| 200% or greater | 1 |
| Sampling Source |  |
| Random digit dialing | 0.51** (0.33,0.79) |
| Address–based | 0.34*** (0.22,0.52) |
| Non–probability | 1 |
| Constant | 1.15 (0.57,2.34) |

Note: * = p < 0.05, ** = p < 0.01, *** = p < 0.001; 1 = reference group; CI = confidence interval.

^a^High SSB consumption refers to 6 or more ounces of SSBs per day.

**Table I. Difference-in-differences of likelihood of changing city of purchase for sugar-sweetened beverages (SSB) pre- and post-tax implementation between San Francisco and San José (n=1,443)**

|  | **Adjusted Odds Ratio**  **(95% CI)** |
| --- | --- |
|  |  |
| **Time** |  |
| Pre–Tax | 1 |
| Year 1 post–tax | 0.66 (0.17,2.56) |
| Year 2 post–tax | 1.60 (0.55,4.66) |
| **City** |  |
| San Francisco | 3.51* (1.24,9.98) |
| San José | 1 |
| **Difference-in-differences** |  |
| City * Year 1 post–tax interaction | 2.11 (0.39,11.53) |
| City * Year 2 post–tax interaction | 0.55 (0.09,3.26) |
| Covariates |  |
| Age, years | 0.99 (0.97,1.00) |
| **Gender** |  |
| Male | 1 |
| Female | 1.22 (0.57,2.59) |
| **Race/Ethnicity** |  |
| Asian or White | 1 |
| Black, Latino, or other race | 0.94 (0.41,2.13) |
| **Education** |  |
| Some college or lower | 1.31 (0.63,2.71) |
| Bachelor’s degree or more | 1 |
| Federal Poverty Level |  |
| Less than 200% | 2.00 (0.86,4.64) |
| 200% or greater | 1 |
| **Sampling Source** |  |
| Random digit dialing | 0.97 (0.44,2.14) |
| Address–based | 0.44 (0.17,1.15) |
| Non–probability |  |
| **Constant** | 0.02*** (0.00,0.09) |

Note: * = p < 0.05, ** = p < 0.01, *** = p < 0.001; 1 = reference group; CI = confidence interval.

^a^High SSB consumption refers to 6 or more ounces of SSBs per day.
